# Links between autism and allergy

**DOI:** 10.1186/1939-4551-8-S1-A17

**Published:** 2015-04-08

**Authors:** Roberto Ronald Cardoso

**Affiliations:** 1 Faciplac Curso De Medicina, Brazil

## Background

Autistic and allergic patients share some immunological patterns. As an example, both have a Th1/Th2 imbalance toward the Th2 pattern. Here we try to demonstrate an immunological similarity: the increased presence of anti-IgE and/or anti-receptor IgE antibodies using the ASST (anti serum skin test) technique.

## Methods

251 individuals participated in this study:

1. 42 with autism without allergies

2. 42 women (mothers of the autistic patients) without autism

3. 104 allergic patients without autism

4. 63 normal health controls

The ASST was performed injecting intradermically 0,05 ml of the each own serum in the volar surface of the forearm. Saline was used as a negative control. After 15 minutes the weals were measured. A result was considered positive when the weal diameter with the serum (this weal was a result of the presence of anti-IgE and/or anti-IgE receptor antibodies) was at least 3 mms greater than that one measured with the saline.

Statistical analysis: chi-squared and Fisher's methods were used to evaluate the results.

## Results

1. from 32 autistics with positive ASST 30 of their mothers were also positives; and from 10 negative autistics 6 of their mothers were negatives. According with the statistical tests we can say that there is an association between the positive results and also with the negative results.

2. when comparing the 63 normal controls with the 42 patients with autism and the 104 allergic individuals without autism it was possible to conclude that the populations with autism and with allergies were not similar to the control group.

## Conclusions

1. the results of the tests in autistics and those ones with their mothers were strongly associated. This result may indicate that in autism genetics the presence of the anti-IgE and/or anti-receptor IgE antibodies can be a marker of heredity, which can be also a marker of autism; 2. the similarity of the results between the positive non-autistic allergics and positive non-allergic autistics shows that the presence of the antibodies may be a common factor between the two conditions, allergy and autism and 3. the presence of the antibodies in autism and allergy occurs differently when compared with the control group.

